# Relative and absolute wealth mobility since birth in relation to health and human capital in middle adulthood: An analysis of a Guatemalan birth cohort

**DOI:** 10.1016/j.ssmph.2021.100852

**Published:** 2021-06-19

**Authors:** Jithin Sam Varghese, Shivani A. Patel, Reynaldo Martorell, Manuel Ramirez-Zea, Aryeh D. Stein

**Affiliations:** a Nutrition and Health Sciences Program, Laney Graduate School, Emory University, Atlanta GA, USA; b Hubert Department of Global Health, Emory University, Atlanta GA, USA; c INCAP Research Center for the Prevention of Chronic Diseases (CIIPEC), Institute of Nutrition of Central America and Panama (INCAP), Guatemala City, Guatemala

**Keywords:** Social mobility, Latent class analysis, Conditional wealth, Life course socio-economic position, BMI, Body Mass Index, HIC, High income country, INCAP, Institute of Nutrition of Central America and Panama, LCA, Latent Class Analysis, LMIC, Low- and middle-income country, MAR, Missing at Random, PCA, Principal Component Analysis, SEP, Socio-economic position, SRQ-20, World Health Organization Self-Reporting Questionnaire-20

## Abstract

**Background:**

Wealth mobility, as both relative (positional) and absolute (material) wealth acquisition, may counteract negative consequences of early life adversities on adult health.

**Methods:**

We use longitudinal data (1967–2018) from the INCAP birth cohort, Guatemala (n = 1386). Using wealth as a measure of socio-economic position, we assess the association of life course relative mobility using latent class analysis and absolute material gains using conditional wealth measures. We estimate associations of wealth mobility with indicators of human capital, specifically height, weight status (BMI in kg/m^2^), psychological distress (WHO SRQ-20 score) and fluid intelligence (Ravens Progressive Matrices score; RPM) in middle adulthood.

**Results:**

We identified four latent classes of relative mobility – Stable Low (n = 498), Stable High (n = 223), Downwardly Mobile (n = 201) and Upwardly Mobile (n = 464). Attained schooling (years) was positively associated with membership in Upwardly Mobile (odds ratio; 1.50, 95%CI: 1.31, 1.71) vs Stable Low, and inversely with membership in Downwardly Mobile (0.65, 95%CI: 0.54, 0.79) vs Stable High. Being Upwardly Mobile (vs Stable Low) was positively associated with height (1.88 cm, 95%CI: 1.04, 2.72), relative weight (1.32 kg/m^2^, 95%CI: 0.57, 2.07), lower psychological distress (−0.82 units, 95%CI: 1.34, −0.29) and fluid intelligence (0.94 units, 95%CI: 0.28, 1.59). Being Downwardly Mobile (vs Stable High) was associated with lower fluid intelligence (−2.69 units, 95%CI: 3.69, −1.68), and higher psychological distress (1.15 units, 95%CI: 0.34, 1.95). Absolute wealth gains (z-scores) from early to middle adulthood were positively associated with relative weight (0.62 kg/m^2^, 95%CI: 0.28, 0.96), lower psychological distress (−0.37 units, 95%CI: 0.60, −0.14) and fluid intelligence (0.50 units, 95%CI: 0.21, 0.79).

**Conclusions:**

Higher attained schooling provided a pathway for upward relative mobility and higher absolute wealth gains as well as protection against downward relative mobility. Upward mobility was associated with lower psychological distress and higher fluid intelligence but also higher weight status.

## Highlights

• Early life wealth and educational attainment are associated with upward wealth mobility, both in relative and absolute terms.
• Upward relative mobility is associated with lower psychological distress, and higher fluid intelligence and BMI.
• Downward relative mobility is associated with lower fluid intelligence, lower height, and higher psychological distress.
• Wealth gains from early to middle adulthood were associated with higher BMI and intelligence and lower psychological distress.

## 1 Introduction

Social mobility, defined as “movements by specific entities between periods in socioeconomic status indicators”, is hypothesized to ameliorate the health effects of early life adversities to which individuals of low socioeconomic position (SEP) are exposed (Behrman et al., 2000; Glymour et al., 2014). Intergenerational persistence, the extent of remaining at the same relative position from birth (parental) to adulthood, may therefore perpetuate health inequities (Marmot & Bell, 2012). Comparison of intergenerational mobility in income and education levels shows that Latin American countries perform worse than their high income country (HIC) counterparts, suggesting that parental SEP matter more for relative mobility in the former, despite intergenerational improvements in standards of living (absolute wealth gains) (Azevedo & Bouillon, 2010; Behrman et al., 2011 ; Narayan et al., 2018; Neidhöfer, 2019). Intergenerational social mobility reflects heritability of ability, early childhood education, social norms, market failures, credit constraints and government expenditure on public services in health and education (Causa & Johansson, 2010). Intergenerational social persistence is correlated with cross-sectional income inequality, such that educational attainment, a key driver of mobility, is often tied to parental income in the absence of government investment (Neidhöfer, 2019).

In Guatemala, GDP per capita (US$, 2010) increased from $1665 in 1965 to $3137 in 2014 with a modest compound annual growth rate of 1.3%, similar to 21 other Latin American countries which experienced a median growth rate of 1.6% (World development indicators. Washington, D.C. :The World Bank). The Gini index, a measure of income inequality, decreased from 0.58 in 1986 (when it was first reported) to 0.48 in 2014 due to increased social assistance spending since early-2000s. Guatemala's Gini index continues to be among the highest in the world (World development indicators. Washington, D.C. :The World Bank). Upward relative mobility and absolute wealth gains can co-occur for individuals in transitioning societies, such as Guatemala, which experienced economic growth and intergeneration gains in attained schooling (Orozco, 2017). However, downward relative mobility only requires an individual's rise in standard of living to be slower than his or her peers, and can occur in the presence of generalized wealth gains at a group level.

Wealth, measured by asset indices, is a robust measure of material capital and socio-economic position in LMIC settings (Filmer & Scott, 2012; Howe et al., 2012). In this paper, we explore wealth mobility as a dimension of social mobility. The association of gains in schooling with wealth mobility and its consequences for health and human capital in later life is underexplored in LMIC settings given the absence of life course data (Azevedo & Bouillon, 2010; Neidhöfer et al., 2018). Additionally, the relative importance of absolute material gains during different life stages for health in later life in LMICs is unknown. To our knowledge, this is the first study which uses prospectively collected life course wealth data to understand the drivers of relative (positional) wealth mobility over the life course in LMIC settings. We also estimate the association of relative wealth mobility and of absolute material gains with health and human capital outcomes in middle adulthood.

## 2 Materials and methods

### 2.1 Study population

The Institute of Nutrition of Central America and Panama (INCAP) conducted a cluster-randomized study in Guatemala in four villages, of which two were assigned to receive Atole and the other two, Fresco. Atole was a higher-energy, protein-containing drink while Fresco was a low-energy drink devoid of protein. Both drinks had similar micronutrient content. Details of the supplementation and cohort characteristics have been described previously (Stein et al., 2008). The study villages were typical of rural settings in Guatemala at the time (Melgar et al., 2020).

All children less than seven years of age living in the study villages were enrolled at study initiation in 1969. Pregnant women were recruited during the study period and newborns were added to the cohort at birth. The cohort of 2392 children born to 816 mothers were followed up to age 7 years, death before study end, or study end in 1977. While the intervention study started in 1969, the preparatory work started in 1966, including household enumeration surveys in the selected study villages in 1967. These surveys were repeated in 1975 and provide household level data that complement the individual participant-level data. Surviving cohort members have been surveyed prospectively since the original trial ended. All participants gave written informed consent before participation in each survey round. We obtained ethical approval for this analysis from the Institutional Review Board of Emory University (Protocol 95960).

Over the period of follow-up the study has experienced attrition, with 1391 of the original participants participating in data collection waves conducted from 2015 to 2018. Our analytic sample consists of 1386 of 1391 participants, after excluding those who lived outside Guatemala (n = 4) or did not report schooling (n = 1). We provide a comparison of the analytic sample with non-participants in Supplementary Table 1.

### 2.2 Data collection and variable specification

#### 2.2.1 Exposure

Information on durables possessed (such as television, radio and refrigerator) and housing characteristics (such as quality of housing construction, house ownership and rooms per member) were collected from cohort households during study waves conducted in 1967 or 1975 (childhood, 0 to 7 years), 1987 (adolescence, 11 to 27 years), 2002 (early adulthood, 25 to 40 years), 2015–16 and 2017–18 (middle adulthood, 37 to 57 years). We use the enumeration wave closest to year of birth of individual participant to describe the household characteristics at birth. For births between 1962 and 1970 (n = 1184) we used the 1967 wave while for births between 1971 and 1977 (n = 1208), we used the 1975 wave. We created cross-sectional asset indices separately for rural (all waves) and urban households (in 2015–16 and 2017–18) at each wave using all available assets and housing characteristics using Principal Component Analysis (PCA). We additionally created a temporally harmonized wealth index based on only the common assets and housing characteristics, the details of which have been reported previously (Varghese et al., 2021). The difference in asset scale between individuals (or over time) derives from differences in asset availability such that the weight computed for an asset is multiplied by the standardized (centered and scaled) value, and then summed across all assets (Filmer & Pritchett, 2001). Our results suggested that the temporally harmonized index was robust to omission/inclusion of assets or study waves, as well as factor extraction procedure (exploratory factor analysis, polychoric PCA, multiple correspondence analysis). We provide the loadings on the first principal component for all asset indices used in Supplementary Table 2.

#### 2.2.2 Outcomes

Height and weight were obtained in 2015–16 (Ford et al., 2016). We computed BMI as weight (in kg) divided by square of height in meters. Ravens Progressive Matrices (RPM), a measure of fluid intelligence, were administered in 2015–16 and if not available, in 2017–18 (Raven, 2000). The WHO Self-Reporting Questionnaire-20 (SRQ-20) was administered in 2017–18 to assess general psychological distress (Beusenberg et al., 1994). Our analytic sample consists of those individuals who reported wealth and an outcome of interest in middle adulthood (participant flow chart in Supplementary Fig 1).

#### 2.2.3 Other variables

Information on maternal characteristics (village, age, height and years of schooling) was collected at enrollment. Information on sex and year of birth was also collected at enrollment. We ascertained exposure to atole during the first 1000 days (roughly the period from conception to 2 years) from date of birth assuming gestation of 266 days. Schooling attainment was collected in 2015–18.

### 2.3 Statistical analysis

#### 2.3.1 Relative wealth mobility

We assessed relative wealth to capture the role of position (rank) along the wealth hierarchy. In the 2015–18 wave, we stratified the sample by urban or rural residence and created stratum-specific tertiles of wealth, since those in urban areas might be wealthier overall than those in rural areas and individuals with the same absolute wealth score might belong to different tertiles of relative wealth.

We classified individuals into latent classes of social mobility using Latent Class Analysis (LCA). LCA empirically identifies discrete latent variables from two or more discrete observed variables (McCutcheon, 1987). The path diagram for LCA is provided in Supplementary Fig 2. LCA was fit with Full Information Maximum Likelihood (FIML) under an assumption of missing at random (MAR). We assessed the number of latent classes using Bayesian Information Criterion (BIC), followed by Bootstrap Likelihood Ratio Test (BLRT), entropy and class sizes (Nylund et al., 2007). We estimate the association of early life characteristics (assignment to supplementation group, exposure in first 1000 days, maternal schooling, year of birth and sex) which are potential predictors of class membership. We additionally used attained schooling as a predictor of class membership given its role in social mobility during transition to adulthood. We fit models with 2 to 5 latent classes. The model fit statistics from different runs are provided in Supplementary Table 3. Based on the results, BIC recommended 3 classes, sample-size adjusted BIC recommended 4 or 5 classes, BLRT recommended 5 classes and entropy recommended 5 classes. However, the size of one of the classes for the 5-class run was 100 (<10% sample). As a compromise between model fit, entropy and class size, we proceeded with 4 latent classes. Results before and after adjusting for clustering by maternal identifier were similar. Hence, we did not adjust for clustering in our reported LCA results.

We estimated the association of class membership with adult outcome variables using linear regression after adjusting for predictors of latent classes (maternal schooling, assignment to supplementation group, exposure during first 1000 days, year of birth, and attained schooling) (Schuler et al., 2014). We assessed probability of classification into the most likely latent class. We assessed for effect modification by sex using F-tests for nested models. We do not quantify the extent of relative mobility, such as using transition matrices.

#### 2.3.2 Absolute wealth gains

We calculated conditional asset index residuals by regressing each asset index measure with all preceding asset index measures for each imputed dataset (Duc, 2019). The conditional residuals are uncorrelated with all previous asset index measures, indicating change beyond the expected wealth index conditional on its predictors. The conditional asset index can be interpreted as capturing absolute wealth gains experienced by an individual or household beyond that experienced by the community on average.

$${\text{CS}}_{\text{iT}}{=\text{S}}_{\text{iT}}-\hat{{\text{S}}_{\text{iT}}}{=\text{S}}_{\text{iT}}-\left[\beta_{\text{0}}+\sum_{\text{t}=1}^{\text{t}=\text{ T}-1}\beta \text{t}\;{\text{S}}_{\text{it}}\right]$$

S_it_ is the asset index measure at time t (ranging from 1 to T-1), $\hat{\text{SiT}}$ is the predicted (expected) asset index measure at time T and CS_iT_ is the conditional asset index for individual I at time T. This approach is an extension of one used previously to study the association of change beyond expected increase in anthropometric measures with adult health outcomes (Adair et al., 2013; Addo et al., 2013, 2015). We used multiple imputation with chained equations (MICE; 10 datasets, 50 iterations) with auxiliary covariates (assignment to supplementation group, exposure in first 1000 days, maternal schooling, birth year, sex, attained schooling, rural residence in adulthood) to impute missing values in our harmonized asset index measures under MAR. Among the analytic sample, 32.7% and 40.7% did not have wealth data in 1987 or 2002 respectively since they did not reside in one of the four study villages at the time of that study wave; 297 did not have asset data in either wave.

We then estimated associations of early life wealth and conditional wealth gains with adult outcomes using linear regression. We adjusted for the auxiliary covariates used in the imputation step.

#### 2.3.3 Sensitivity analysis

We repeated both analyses using generalized estimating equations with inverse probability of censoring weights for being alive at time of outcome assessment and for participating in adulthood but not reporting outcome of interest. We also compared results without the use of auxiliary covariates in multiple imputation for absolute wealth gains. We compared results of association of wealth mobility with SRQ-20 after treating it as counts of symptoms of psychological distress under a Poisson distribution. We compare coefficients for absolute wealth gains by year of data collection of early life wealth (1967 vs 1975).

All analysis was carried out using Mplus 7.4 and R 3.5.1 using mice 3.4.0 and tidyverse 1.3.0.

## 3 Results

The analytic sample consisted of 1386 individuals from the 2021 who were alive in 2015–18. Participants were predominantly female (55.6%) with a median of 1 year of maternal schooling and 5 years of own attained schooling.

### 3.1 Latent class analysis of relative wealth mobility

**Fig. 1:**
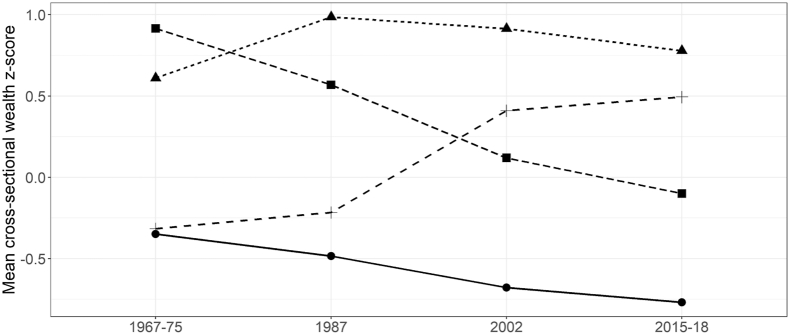
Mean wealth over time by latent class of relative wealth mobility in INCAP study cohort, Guatemala (n = 1, 386). Mean wealth z-score at different study waves by latent class of relative wealth mobility.

Based on their aggregate compositions, we identified 4 latent classes of relative mobility – Stable Low (n = 498), Stable High (n = 223), Downwardly Mobile (n = 201) and Upwardly Mobile (n = 464). We display the mean wealth z-scores over time by latent class of relative wealth mobility in Fig. 1. Classification probabilities for most likely class membership suggest higher proportion of potential misclassification for ‘Stable High’ (25%), ‘Downwardly Mobile’ (37%) and ‘Upwardly Mobile’ (23%) classes (Supplementary Table 4) relative to ‘Stable Low’ class (14%). Individuals assigned to Stable Low showed a pattern in which the proportion of those classified into the lowest wealth tertile increased over time (Supplementary Table 5). Among those assigned to Downwardly Mobile, the proportion of those classified into the highest wealth tertile decreased, while the proportion of those classified into medium wealth tertile increased. Among those assigned to Upwardly Mobile, the proportion of those classified into high wealth tertile was low at baseline and increased over time. The proportion of those classified into high wealth tertile was high in 1967–75 and increased over time among those assigned to Stable High.

We provide descriptive statistics by class membership in Table 1. The Stable High class had higher median maternal schooling [2 y, IQR: 1–3] and attained schooling [10 y, IQR: 6–12] in comparison to other classes. The Downwardly Mobile class had higher median maternal schooling (y, 2 vs 0) and attained schooling (y, 6 vs 2) relative to Stable Low. The Upwardly Mobile class, in contrast to Stable Low, had higher attained schooling [y; 6 vs 2] and were less likely to be female (52% vs 55%), but had similar maternal schooling.

**Table 1:** Descriptive characteristics by most likely latent class membership among participants in middle adulthood (n = 1386).

	Pooled	Stable Low (n = 498)	Stable High (n = 223)	Downwardly Mobile (n = 201)	Upwardly Mobile (n = 464)
Atole supplemented	53%	55%	57%	49%	50%
Exposure during first 1000 days	41%	41%	48%	37%	38%
Atole x 1000 days	21%	23%	28%	13%	20%
Maternal Schooling (y)^1^	1 [0, 2]	0 [0, 2]	2 [1, 4]	2 [0, 3]	0 [0, 2]
Year of Birth	1970 [1967, 1974]	1970 [1967, 1973]	1971 [1968, 1973]	1970 [1967, 1974]	1970 [1966, 1974]
Female	56%	55%	57%	63%	52%
Attained Schooling (y)	5 [2, 6]	2 [0, 4]	10 [6, 12]	6 [3, 6]	6 [3, 6]
Residing in urban area in 2015–18	28%	24%	40%	28%	28%
Outcomes in adulthood^2^ (2015–18)					
Adult height (cm)	156 ± 8.3	155 ± 7.8	159 ± 8.2	156 ± 8.4	157 ± 8.3
Body mass index (kg/m^2^)	28.2 ± 5.1	27.6 ± 5.4	28.7 ± 4.9	28.3 ± 5.2	28.6 ± 4.7
WHO SRQ-20	3 [1, 6]	3 [1, 7]	2 [1, 5]	4 [1, 7]	2 [1, 5]
Ravens progressive matrices	16.4 ± 5.6	14.1 ± 3.9	21.3 ± 6.2	15.5 ± 5.5	16.8 ± 5.3

Continuous variables are displayed as mean ± standard deviation (if normally distributed) or median [25th percentile, 75th percentile]. Categorical variables are displayed as percentage (%). ^1^ Maternal schooling is available for 1345 individuals; ^2^ BMI data are available for 1145 individuals; SRQ-20 data are available for 1264 individuals; Ravens data are available for 1331 individuals.

Multinomial logistic regression coefficients predicting class membership are presented in Table 2. Cohort members in the Stable Low class had lower attained schooling than individuals in the other three classes (each p < 0.001) and lower maternal attained schooling than those in the Stable High or Downwardly Mobile classes (both p < 0.001). Relative to individuals in the Stable High class, those in the Downwardly Mobile class had lower attained schooling (p < 0.001).

**Table 2:** Odds ratios from multinomial regression predicting relative mobility class membership in Latent Class Analysis (n = 1386).

*Relative to Stable Low*	Stable Low (n = 498)	Stable High (n = 223)	Downwardly Mobile (n = 201)	Upwardly Mobile (n = 464)
Maternal Schooling (y)^1^	Ref: 1.00	1.76 (1.19, 2.63)	1.49 (1.13, 1.96)	1.02 (0.85, 1.21)
Year of Birth	Ref: 1.00	0.9 (0.81, 1)	0.99 (0.88, 1.10)	0.96 (0.89, 1.03)
Sex = Male	Ref: 1.00	0.46 (0.22, 0.92)	0.56 (0.26, 1.24)	0.79 (0.52, 1.21)
Attained Schooling (y)	Ref: 1.00	2.02 (1.65, 2.47)	1.32 (1.12, 1.55)	1.50 (1.31, 1.71)
*Relative to Stable High*				
Maternal Schooling (y)^a^	0.57 (0.38, 0.84)	Ref: 1.00	0.85 (0.5, 1.42)	0.58 (0.41, 0.8)
Year of Birth	1.11 (1.00, 1.23)	Ref: 1.00	1.09 (0.94, 1.28)	1.06 (0.96, 1.18)
Sex = Male	2.20 (1.08, 4.45)	Ref: 1.00	1.24 (0.42, 3.7)	1.74 (0.84, 3.6)
Attained Schooling (y)	0.50 (0.40, 0.61)	Ref: 1.00	0.65 (0.54, 0.79)	0.74 (0.63, 0.88)

Adjusted for supplementation and exposure in first 1000 days.

a Imputed with village mean for 41 observations to avoid dropping those participants since it was an exogenous variable.

### 3.2 Association of latent classes with health outcomes

**Fig. 2:**
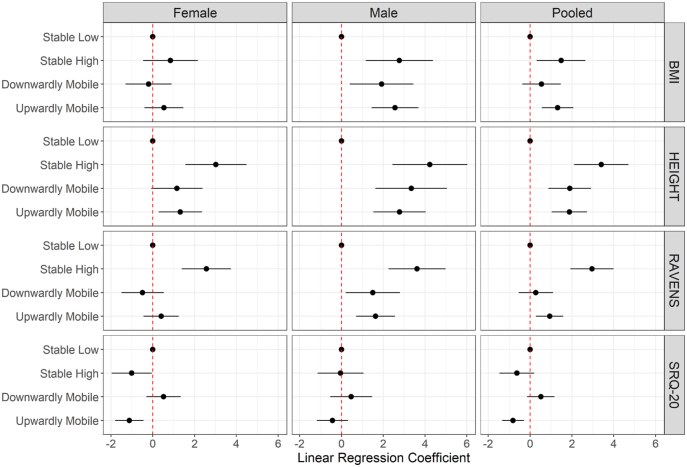
Coefficients from multivariable linear regression for association of class membership with measures^1^ of health and human capital in middle adulthood stratified (37 to 57 years).Assessment of heterogeneity by sex was carried out using F-tests for nested models. Heterogeneity by sex was present for association of BMI and classes (F = 3.6, p = 0.01). F statistic and p-value were not significant for Height (F = 1.9, p = 0.12), Ravens (F = 2.5, p = 0.06) and SRQ-20 (F = 1.35, p = 0.25). All models are adjusted for predictors of latent classes: maternal schooling, assignment to supplementation group, exposure during first 1000 days, year of birth, and attained schooling. BMI data available for 1138 individuals; Height data available for 1144 individuals; SRQ-20 data available for 1264 individuals; Ravens data available for 1330 individuals.

After adjusting for predictors of latent classes, relative to Stable Low, Upwardly Mobile had higher BMI (1.32 kg/m^2^, 95% CI: 0.57, 2.07), lower SRQ-20 (−0.82 units, 95% CI: 1.34, −0.29) and higher RPM (0.94 units, 95% CI: 0.28, 1.59). Stable High, relative to Stable Low, and higher RPM (2.96, 95% CI: 1.93, 3.99). Downwardly mobile, relative to Stable Low, had higher SRQ-20 score (0.51 units, 95% CI: 0.14, 1.17). Relative to Stable Low, the other classes were taller. Being Downwardly Mobile (vs Stable High) was associated with lower RPM (−2.69 units, 95% CI: 3.99, −1.93), and higher SRQ-20 (1.15 units, 95% CI: 0.34, 1.95). We observed heterogeneity by sex for association of latent class membership with BMI (F = 3.6, p = 0.01). We did not find statistically significant (at p = 0.05) heterogeneity by sex for the other outcomes. Results for pooled and sex-stratified analysis are presented in Table 3 and Fig. 2 respectively.

**Table 3:** Coefficients from multivariable linear regression for association of class membership with measures^a^ of health and human capital in middle adulthood (37 to 57 years).

*Relative to Stable Low*	Height (cm)	Body mass index (kg/m^2^)	WHO SRQ-20	Ravens progressive matrices
Stable Low	Ref = 0.00	Ref = 0.00	Ref = 0.00	Ref = 0.00
Stable High	3.41 (2.11, 4.7)	1.48 (0.32, 2.65)	−0.63 (−1.47, 0.2)	2.96 (1.93, 3.99)
Downwardly Mobile	1.90 (0.87, 2.92)	0.55 (−0.37, 1.47)	0.51 (−0.14, 1.17)	0.27 (−0.55, 1.10)
Upwardly Mobile	1.88 (1.04, 2.72)	1.32 (0.57, 2.07)	−0.82 (−1.34, −0.29)	0.94 (0.28, 1.59)
*Relative to Stable High*				
Stable Low	−3.41 (−4.7, −2.11)	−1.48 (−2.65, −0.32)	0.63 (−0.2, 1.47)	−2.96 (−3.99, −1.93)
Stable High	Ref = 0.00	Ref = 0.00	Ref = 0.00	Ref = 0.00
Downwardly Mobile	−1.51 (−2.77, −0.24)	−0.94 (−2.07, 0.20)	1.15 (0.34, 1.95)	−2.69 (−3.69, −1.68)
Upwardly Mobile	−1.53 (−2.63, −0.42)	−0.16 (−1.16, 0.83)	−0.18 (−0.9, 0.53)	−2.02 (−2.9, −1.15)

All models are adjusted for predictors of latent classes: maternal schooling, assignment to supplementation group, exposure during first 1000 days, year of birth, and attained schooling.

a Height data available for 1144 individuals; BMI data available for 1138 individuals; SRQ-20 data available for 1264 individuals; Ravens data available for 1330 individuals.

### 3.3 Association of absolute wealth gains with health outcomes

After adjusting for early life characteristics and rural residence in adulthood, each 1 unit gain in harmonized wealth from adolescence to early adulthood (1987 to 2002; ‘Conditional Wealth 2002’) was associated with higher BMI (0.30, 95% CI: 0.05, 0.66), lower SRQ-20 (−0.28, 95% CI: 0.50, −0.05) and higher RPM score (0.33, 95% CI: 0.04, 0.61). Each 1 unit gain in absolute wealth from early adulthood to middle adulthood (2002 to 2015–18; ‘Conditional Wealth 2015–18’) was associated with higher BMI (0.62, 95% CI: 0.28, 0.96), lower SRQ-20 (−0.37, 95% CI: 0.60, −0.14) and higher RPM score (0.50, 95% CI: 0.21, 0.79). Coefficients for the regressions are presented in Table 4.

**Table 4:** Coefficients from multivariable linear regression for association of harmonized wealth score gains over the life course with measures^a^ of health and human capital in middle adulthood.

	Body mass index (kg/m^2^)	WHO SRQ-20	Ravens Progressive Matrices
Wealth in 1967–75	0.05 (−0.24, 0.33)	−0.01 (−0.21, 0.20)	0.10 (−0.16, 0.35)
Conditional Wealth 1987	0.07 (−0.21, 0.35)	0.08 (−0.10, 0.26)	0.40 (0.19, 0.62)
Conditional Wealth 2002	0.30 (−0.05, 0.66)	−0.28 (−0.50, −0.05)	0.33 (0.04, 0.61)
Conditional Wealth 2015–18	0.62 (0.28, 0.96)	−0.37 (−0.60, −0.14)	0.50 (0.21, 0.79)

Wealth is estimated as a temporally harmonized index of assets and housing characteristics; all associations (change per 1 unit in wealth) are adjusted for maternal schooling, assignment to supplementation group, exposure during first 1000 days, year of birth, sex, attained schooling and rural residence in adulthood..

a BMI data available for 1138 individuals; SRQ-20 data available for 1264 individuals; Ravens data available for 1330 individuals.

### 3.4 Sensitivity analysis

We reported the results for our sensitivity analysis for missingness in Supplementary Table 6 and Supplementary Table 7. Our results were similar for both analyses across all outcomes of interest with and without inverse probability of censoring weights for being alive and for reporting outcome of interest in adulthood. Our results were also similar before and after we excluded auxiliary covariates (atole supplementation, exposure during first 1000 days, maternal schooling, sex, birth year and attained schooling) from multiple imputation of wealth in 1987 and 2002. Results using zero-inflated Poisson Regression for association of relative wealth mobility and absolute wealth mobility with SRQ-20 displayed similar results (Supplementary Table 8). Association of absolute wealth gains with health in middle adulthood were similar between strata of year of early life wealth data collection (1967 for pre-1971 born, 1975 for post-1971 born) except for early life wealth and conditional wealth in 1987 (early life to adolescence) for SRQ-20 and RPM (Supplementary Table 9).

## 4 Discussion

Our results suggest that higher attained schooling was associated with upward relative mobility, compared to those who remained persistently low, in this cohort followed prospectively for over 50 years. The findings are consistent with results from both HIC and LMIC settings (Behrman et al., 2011; Krzyzanowska & Mascie-Taylor, 2013). Our results also suggest that higher attained schooling was associated with lower odds of downward relative mobility, relative to being persistently high. Upward relative mobility was associated with higher BMI, lower psychological distress and higher fluid intelligence in middle adulthood. Relative to those who experienced persistently low relative wealth (Stable Low class), downward relative mobility (Downwardly Mobile class) was associated with higher fluid intelligence and psychological distress scores but similar BMI. Absolute wealth gains from adolescence to early adulthood and from early adulthood to middle adulthood were associated with all health outcomes in middle adulthood. Wealth in early life was not associated with health in middle adulthood after adjusting for covariates and later life measures of wealth.

### 4.1 Determinants of relative mobility

Results from this analysis suggests a persistence of social class, with schooling as a key enabler of mobility. The Stable Low and Upwardly Mobile classes, which were otherwise similar, differed markedly in attained schooling (2 y vs 6 y). This potentially explains gains in fluid intelligence (which peaks in young adulthood) and protection against psychological distress in middle adulthood. Similarly, the Stable High and Downwardly Mobile classes differed in schooling attainment despite being similar in several early-life characteristics including maternal schooling. Attained schooling for our study population peaked in adolescence. Therefore, any schooling-related gaps in human capital and wealth accumulation are unlikely to close over time, highlighting the importance of early life investments. The proportion of females in the Stable High class (57%) was lower than in the Downwardly Mobile class (63%). However, there was no association of sex with class membership after adjusting for attained schooling and other early life characteristics, suggesting a potentially leveling role of schooling in reducing sex disparities. We additionally note that wealth mobility, as defined by relative wealth trajectories, is a zero-sum game. The association of relative wealth and health is hypothesized to be mediated by preferential access to resources and psychosocial stressors which may manifest itself in absolute health inequities. Alternative formulations that consider absolute wealth gains and relational positioning could be useful in examining this issue.

Previous research from this cohort has suggested moderate benefits of nutritional supplementation in early childhood on attained schooling (1.2 grades) for women as well as on higher reading comprehension and non-verbal cognitive ability for both men and women (Maluccio et al., 2009). Combined interventions of nutritional supplementation and early childhood education could yield substantial benefits (Black et al., 2017; Larson & Yousafzai, 2017; Yousafzai et al., 2016). Additionally, gains from early life interventions could be lost if structural investments for upward social mobility (such as infrastructure) and safety nets protecting against downward mobility (such as ensuring quality and performance of subsequent schools) are absent, as shown in evaluations of the US Head Start program, reinforcing the need for continued interventions through the life course (Zhai et al., 2012).

### 4.2 Psychosocial and neo-materialistic frameworks

The scenario of rising living standards with persistence of relative deprivation presents an opportunity to examine the roles of psychosocial and neo-materialistic frameworks of SEP on health and well-being (Supplementary Fig 3). An individual in lower relative SEP experiences higher exposure to environmental and structural stressors and lower control over life circumstances. This could result in chronic activation of the neuroendocrine and endocrine pathways or adoption of harmful coping behaviors (such as drug use and binge drinking) (Havranek et al., 2015; Tian et al., 2019). Therefore, individuals experiencing upward relative mobility may also experience higher control over their circumstances, resulting in lower distress and higher well-being as shown in our study (Marmot & Wilkinson, 2001).

The neo-materialist framework suggests that gains in material capital, at a societal or community level, could lead to better health via accumulation of material and psychological resources as well as investments in preventive and social services for well-being (Lynch et al., 2000). This association of material capital with health is combined result of structural investments in infrastructure, higher standards of living and lower exposure to stressors such as crime and pollution. As shown in our study, individuals who experienced absolute gains in wealth had higher mental and socio-emotional well-being. Our results do not speak to psychosocial versus material pathways, but rather address whether relative material mobility relates to health and human capital.

### 4.3 Association of mobility with health and human capital

Human capital consists of both endowments fixed in early life or adolescence (such as height and perhaps cognition) as well as mutable characteristics like education and skills (Oakes & Rossi, 2003). At the time that our cohort were in school, opportunities were limited. However, as a result of national literacy campaigns in Guatemala, the years of attained schooling more than tripled from 1967 to 2015 (Melgar et al., 2020). Human capital indicators such as height and cognition tend to be results of early life investments in nutrition and early child development (Black et al., 2017). The association of wealth with fluid intelligence is susceptible to reverse causation, as individuals with higher intelligence in our study could have experienced upward relative mobility or absolute gains in wealth. High childhood cognition (from genetic endowments and early childhood education) is associated with upward social mobility, potentially through higher educational attainment or utilization of parental resources, and subsequent acquisition (or prevent deterioration) of cognitive skills in adulthood (Elbejjani et al., 2017; McGue et al., 2020; Oi & Haas, 2019).

Physical and mental health were associated with social mobility. Our results show a positive gradient between BMI and wealth, an association observed in several LMICs undergoing the nutrition transition (Jaacks et al., 2019). Nationally in 2016, the mean adult BMI was 25.8 kg/m^2^ among men and 27.5 kg/m^2^ among women, similar to other LMIC countries in the region (Worldwide trends in body-, 2017). Our analysis adds to the evidence that upward social mobility (in both absolute and relative terms) is associated with lower psychological distress (Gaydosh et al., 2018). Our results also support the hypothesis that cumulative deprivation and loss of wealth are associated with greater risk of mental health issues (Melchior et al., 2018; Nicklett & Burgard, 2009). A country's or community's stage of obesity transition was an effect modifier for association of persistently high SEP or upward mobility with adiposity such that in many LMIC settings upward mobility was associated with higher adiposity while results were dependent on race and community in HIC settings (Gaydosh et al., 2018; Jaacks et al., 2019; Lartey et al., 2019; Miller et al., 2020). Both relative mobility and household income were shown to be inversely associated with rates of depression in HIC and LMIC settings (Melchior et al., 2018; Nicklett & Burgard, 2009; Tiffin et al., 2005; Ward et al., 2016).

### 4.4 Strengths and limitations

Our analysis represents the longest follow-up for a prospective birth cohort in a low- or middle-income country setting. Data on assets and housing characteristics were collected at each wave for participants residing in or near their birth village, and for those participants who migrated elsewhere in Guatemala in 2015–16 and 2017–18. Despite this, our analysis has certain limitations. Firstly, asset data was not available for cohort members who resided outside study villages in 1987 and 2002. We assumed missing at random while proceeding with our analysis (FIML for relative wealth mobility and multiple imputation for absolute wealth gains). Our analysis with and without auxiliary covariates for imputation showed similar results. Second, the study experienced attrition such that of the 2021 cohort members who were known to be alive in 2015–18, 1391 participated. A prior analysis comparing participants with non-participants showed that participants were more likely to be female and had 1 year lower median maternal schooling (Varghese et al., 2021). Third, since our study villages were representative of rural non-Indigenous Guatemalans in 1967, we cannot generalize findings to those who were born in urban areas or those belonging to Indigenous communities (Poder & He, 2015). High levels of intergenerational persistence of poverty and affluence, marked by ethnic (indigenous and ladino) and regional (urban and rural) differences, continue to be observed in Guatemala. A recent study shows that schooling and wage inequalities between ladino and indigenous populations persist, although improvements in educational outcomes have led to convergence in wages over time (Canelas & Gisselquist, 2018). Given the share of average tax burden, personal income taxes and social spending at 13.0% (2013), 0.4% (2013) and 7.4% (2015) of GDP respectively, among the lowest in Latin America, tax-based redistribution is limited (Cabrera et al., 2015). Moreover, our measures of wealth mobility are constrained by the observed sample and therefore, do not capture the extent of possible mobility in Guatemala. Fourth, our analysis looks only at wealth mobility in terms of asset indices. Though other dimensions of social mobility (income, savings, occupational prestige, education, height) could present different results, these measures of SEP are often highly correlated. Additionally, the absolute wealth gains observed in our study could be the result of local structural investments (such as sewage or garbage disposal) or asset purchases by the household that might not be captured by our instrument. Asset-based wealth measures rely on the inherent assumption that accumulation of household assets and housing characteristics is a proxy for wealth. Our study is also different from wealth mobility studies in high-income settings that rely on estate inventory reports, gifts or inheritances and financial assets (Adermon et al., 2018; Pfeffer & Killewald, 2018). Finally, our latent classes for relative mobility indicated potential misclassification with moderate entropy (0.62). An ideal entropy of 1.00 would have indicated perfect separation between latent classes.

## 5 Conclusion

Future research could look at pathways through which wealth operates in different stages of the life course to affect health in middle adulthood. Possible mechanisms include transitioning dietary and activity patterns which lead to higher adiposity as well as pursuit of leisure and lower exposure to stressful experiences (Popkin, 2015). Given that economic disparities have been amplified all over the world in recent months by SARS-CoV-2, research on long-term implications of downward relative mobility and loss in absolute wealth is also important (Dorn et al., 2020; Slotman, 2020). In the absence of intergenerational social mobility, a combination of labor market dynamics and disproportionate gains in wages from higher skills (from investments in human capital by the rich) can amplify inequality (Azevedo & Bouillon, 2010). This is especially crucial in LMIC settings where exposure to financial adversities and income inequality in childhood might compromise early life growth and developmental trajectories potentially setting today's children on a course of cumulative deprivation and restricted social mobility (Neidhöfer, 2019). Previous research from this population showed that association of parental resources with overall schooling outcomes decreased over time but efforts in reduction of gender disparities in schooling lag behind other Latin American countries (Yount et al., 2013). Policies aimed strengthening existing social safety nets and developing remedial mechanisms for lost opportunity ought to be prioritized.

## Declaration of competing interest

None.

## Availability of data and materials

The datasets generated and/or analysed during the current study are not publicly available. There are ethical or legal restrictions on sharing a de-identified data set. We cannot anonymize the data from this cohort as all individuals come from one of four previously named villages and hence are readily re-identifiable once their demographic characteristics are known. We will not post data to a public archive, but we will make a replication data set available to bona fide researchers who agree to sign an LDUA and are covered under an IRB. Please contact the Research Center for the Prevention of Chronic Diseases (CIIPEC) at the Institute of Nutrition of Central America and Panama for requests. The local data protection manager is Dina Roche (email: droche@incap.int; phone: +502 5499 7220). The code is available at https://github.com/jvargh7/incap-social-mobility.
