# Supplementary material for: Relative and absolute wealth mobility since birth in relation to health and human capital in middle adulthood: An analysis of a Guatemalan birth cohort
Source: SSM Popul Health. 2021 Jun 19;15:100852. doi: 10.1016/j.ssmph.2021.100852 (PMC8242036; doi:10.1016/j.ssmph.2021.100852)
Supplement: Multimedia component 1 [file mmc1.docx]

**Table of Contents**

[Supplementary Fig 1. Flowchart of participants reporting wealth and health in middle adulthood in INCAP Longitudinal Study 1969-2018 3](#_Toc73017681)

[Supplementary Fig 2. Structural equation model for Latent Class Analysis for relative wealth mobility 4](#_Toc73017682)

[Supplementary Fig 3. Neo-materialistic and psychosocial frameworks for association of SES and health 5](#_Toc73017683)

[Supplementary Table 1. Early life characteristics of cohort members by participation status in adulthood (n = 2392) 6](#_Toc73017684)

[Supplementary Table 3. Model statistics for latent class analysis for relative wealth mobility (n = 1,387) 9](#_Toc73017685)

[Supplementary Table 4. Classification probabilities for most likely class membership by Latent Class 10](#_Toc73017686)

[Supplementary Table 5. Membership in latent classes of relative wealth mobility by relative wealth tertiles over the life course (n= 1387) 11](#_Toc73017687)

[Supplementary Table 6. Coefficients from regression with inverse probability weights^1^ for association of class membership with measures^2^ of health and human capital in middle adulthood (2015-18) 12](#_Toc73017688)

[Supplementary Table 7. Coefficients from regression without auxiliary covariates in multiple imputation and with inverse probability weights for association of life course wealth gains with health outcomes in middle adulthood 13](#_Toc73017689)

[Supplementary Table 8. Coefficients from zero-inflated poisson regression for association of wealth mobility with WHO SRQ-20 15](#_Toc73017690)

[Supplementary Table 9. Coefficients for association of absolute wealth gains with health in middle adulthood after stratifying by year at which early life wealth data was collected (n = 1386) 16](#_Toc73017691)

# Supplementary Fig 1. Flowchart of participants reporting wealth and health in middle adulthood in INCAP Longitudinal Study 1969-2018

**
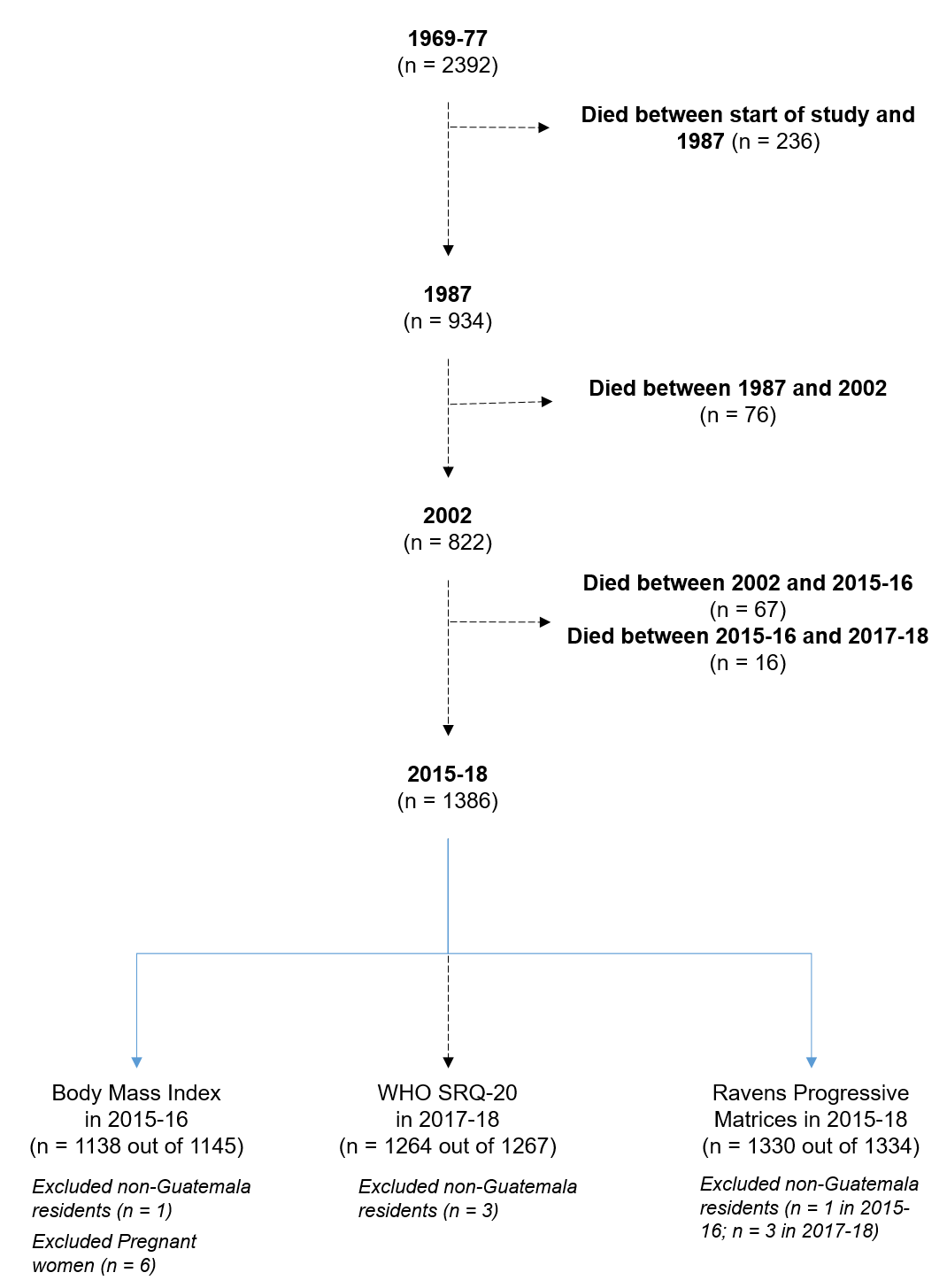
**

#
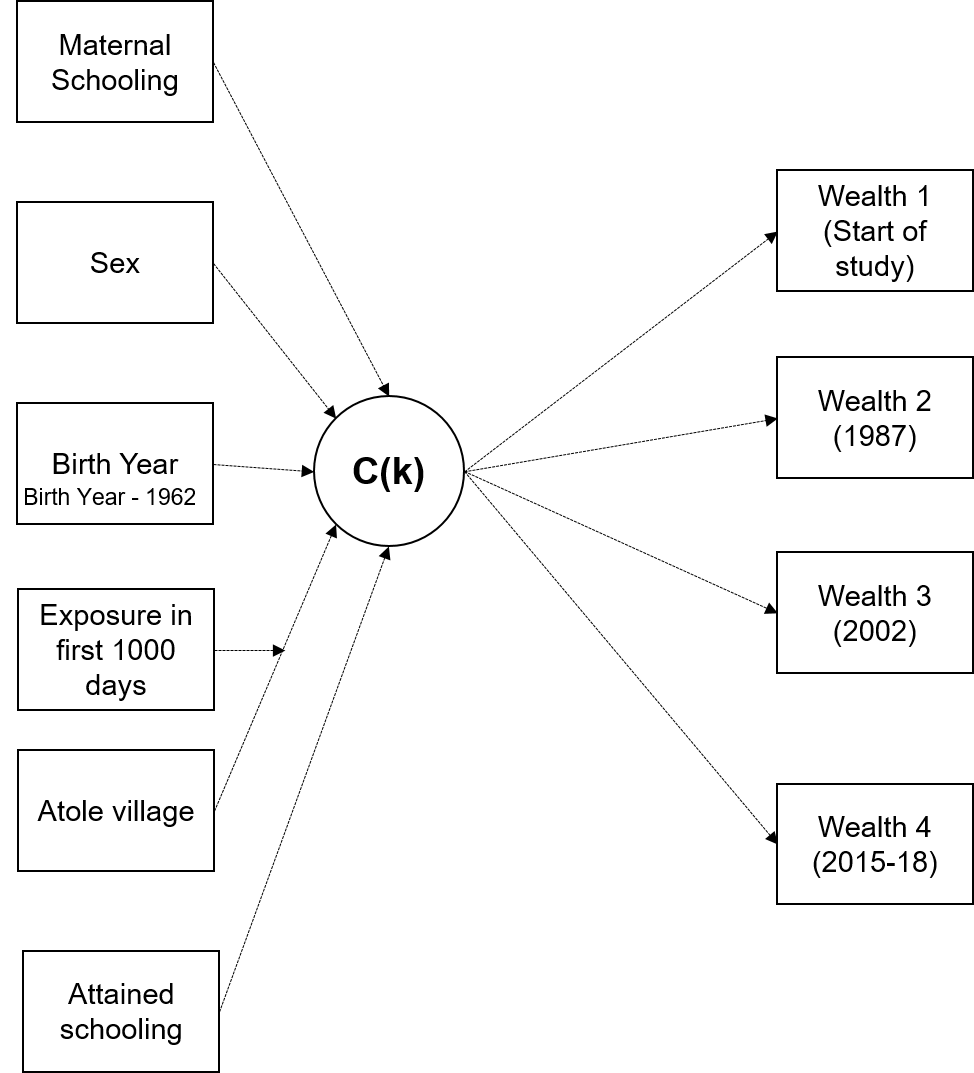
Supplementary Fig 2. Structural equation model for Latent Class Analysis for relative wealth mobility

# Supplementary Fig 3. Neo-materialistic and psychosocial frameworks for association of SES and health

**
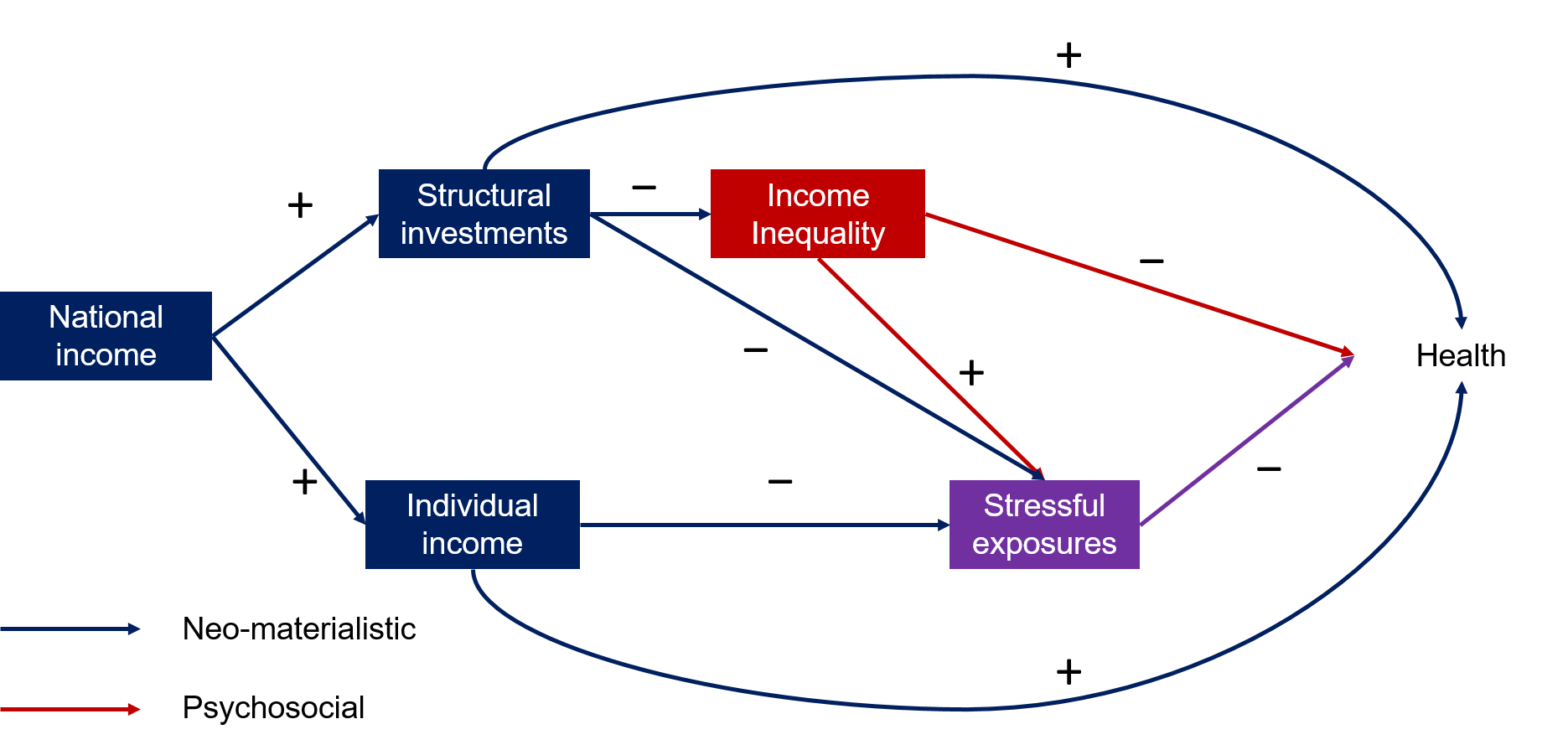
**

# Supplementary Table 1. Early life characteristics of cohort members by participation status in adulthood (n = 2392)

|  | **2015-16** |  |  | **2017-18** |  |  |
| --- | --- | --- | --- | --- | --- | --- |
|  | **Participated**  **(n = 1161)** | **Did not participate**  **(n = 862)** | **Died prior to study wave**  **(n = 369)** | **Participated**  **(n = 1265)** | **Did not participate**  **(n = 742)** | **Died prior to study wave**  **(n = 385)** |
| Maternal schooling (years) | 1 (0,2) | 1 (0,2) | 0 (0,2) | 1 (0,2) | 1 (0,2) | 0 (0,2) |
| Maternal age (years) | 27.1±7.2 | 26.7±7.1 | 27.5±7.7 | 27.0±7.1 | 26.7±7.3 | 27.5±7.7 |
| Harmonized wealth in 1967-75 | -0.2±1.5 | -0.2±1.4 | -0.3±1.4 | -0.3±1.4 | -0.1±1.4 | -0.3±1.4 |
| Birth year | 1970  (1966, 1973) | 1971  (1967, 1974) | 1972  (1968,74) | 1970  (1967, 1973) | 1971  (1967, 1974) | 1971  (1968, 1974) |
| Male | 42% | 58% | 60% | 48% | 53% | 58% |
| Atole supplemented | 54% | 50% | 56% | 53% | 52% | 56% |
| Exposure during first 1000 days | 41% | 43% | 46% | 41% | 43% | 44% |
| Atole x 1000 days | 22% | 22% | 26% | 21% | 24% | 25% |

**Supplementary Table 2. Loadings for harmonized asset index and cross-sectional indices with all assets for INCAP cohort**

|  | **Harmonized** | **Cross-sectional** | | | | **Rural** | | | **Urban** | | |
| --- | --- | --- | --- | --- | --- | --- | --- | --- | --- | --- | --- |
|  |  | **1967** | **1975** | **1987** | **2002** | | **2015-16** | **2017-18** | | **2015-16** | **2017-18** |
| Radio | -0.05 | 0.25 | 0.25 | 0.25 | 0.02 | | 0.01 | 0.02 | | 0 | 0.01 |
| Record Player | 0.00 | - | 0.18 | 0.24 | - | | - | - | | - | - |
| Sewing Machine | 0.12 | - | 0.26 | 0.19 | 0.2 | | 0.16 | 0.14 | | 0.16 | 0.11 |
| Refrigerator | 0.28 | 0.24 | 0.28 | 0.2 | 0.34 | | 0.3 | 0.29 | | 0.28 | 0.26 |
| Television | 0.34 | - | 0.17 | 0.35 | 0.26 | | 0.25 | 0.22 | | 0.27 | 0.22 |
| Bicycle | 0.21 | 0.18 | 0.17 | 0.2 | 0.18 | | 0.1 | 0.16 | | 0.07 | 0.06 |
| Motorcycle | 0.17 | - | 0.19 | 0.08 | 0.08 | | 0.2 | 0.11 | | 0.20 | 0.09 |
| Automobile | 0.18 | - | 0.14 | 0.1 | 0.21 | | 0.24 | 0.24 | | 0.23 | 0.25 |
| Owns land | 0.07 | - | 0.05 | 0.2 | 0.14 | | 0.09 | 0.16 | | 0.09 | 0.14 |
| Owns house | 0.03 | 0.02 | 0.07 | 0.19 | 0.17 | | 0.11 | 0.15 | | 0.12 | 0.12 |
| Rooms per member | 0.17 | 0.24 | 0.21 | 0.04 | 0.14 | | 0.15 | 0.17 | | 0.13 | 0.15 |
| High quality floor | 0.33 | 0.33 | 0.33 | 0.29 | 0.21 | | 0.21 | 0.17 | | 0.24 | 0.17 |
| High quality roof | 0.20 | 0.32 | 0.28 | 0.20 | 0.07 | | 0.05 | 0 | | 0.08 | 0.01 |
| High quality walls | 0.26 | 0.42 | 0.35 | 0.24 | 0.15 | | 0.11 | 0 | | 0.14 | 0.11 |
| Separate kitchen | 0.20 | 0.38 | 0.33 | 0.21 | 0.07 | | 0.08 | 0.15 | | 0.06 | 0.12 |
| Formal cooking medium | 0.28 | 0.34 | 0.31 | 0.22 | 0.22 | | 0.18 | 0.17 | | 0.2 | 0.21 |
| Sanitary installation | 0.33 | 0.35 | 0.26 | 0.23 | 0.15 | | 0.11 | 0.1 | | 0.13 | 0.07 |
| Electricity | 0.33 | 0.04 | 0.1 | 0.27 | 0.2 | | 0.17 | 0.14 | | 0.17 | 0.04 |
| Improved water source | 0.32 | 0.13 | 0.05 | 0.07 | - | | 0.07 | 0.13 | | 0.10 | 0.08 |
| Hand grinder |  | - | - | 0.13 | 0.04 | | - | - | | - | - |
| Electric Iron |  | - | - | 0.34 | 0.24 | | - | - | | - | - |
| Pigs |  | - | - | 0.11 | 0.04 | | 0.00 | -0.08 | | 0.01 | -0.02 |
| Poultry |  | - | - | 0.10 | 0.10 | | 0.02 | -0.09 | | 0.03 | -0.09 |
| Improved sewage system |  | - | - | 0.00 | 0.13 | | 0.12 | 0.2 | | 0.1 | 0.19 |
| VCR |  | - | - | - | 0.25 | | 0.23 | 0.2 | | 0.23 | 0.19 |
| Music system |  | - | - | - | 0.30 | | 0.22 | 0.2 | | 0.21 | 0.15 |
| Computer |  | - | - | - | 0.14 | | 0.25 | 0.28 | | 0.21 | 0.28 |
| Telephone |  | - | - | - | 0.21 | | 0.19 | 0.14 | | 0.21 | 0.12 |
| Cable TV |  | - | - | - | 0.07 | | 0.24 | 0.21 | | 0.22 | 0.21 |
| Microwave |  | - | - | - | 0.18 | | 0.26 | 0.25 | | 0.23 | 0.27 |
| Blender |  | - | - | - | 0.31 | | 0.28 | 0.22 | | 0.26 | 0.24 |
| Washing machine |  | - | - | - | - | | 0.2 | 0.28 | | 0.2 | 0.27 |
| Cellphone |  | - | - | - | - | | 0.19 | 0.08 | | 0.21 | 0.11 |
| Ipod |  | - | - | - | - | | 0.13 | 0.12 | | 0.13 | 0.22 |
| Public garbage system |  | - | - | - | - | | 0.13 | 0.24 | | 0.13 | 0.19 |
| Internet |  | - | - | - | - | | - | - | | 0.13 | 0.26 |
| Direct TV/Netflix |  | - | - | - | - | | - | - | | 0.05 | 0.17 |
| **% Variance explained by PC1** | **32.4** | **19.5** | **17.5** | **16.5** | **13.7** | | **14.9** | **14.9** | | **16.3** | **17.1** |
| **Households (n)** | **4959** | **547** | **755** | **617** | **820** | | **766** | **816** | | **307** | **329** |

# Supplementary Table 3. Model statistics for latent class analysis for relative wealth mobility (n = 1,386)

| **K** | **Class sizes** | **BIC** | **aBIC** | **BLRT P-value** | **Entropy** |
| --- | --- | --- | --- | --- | --- |
| 2 | 720,666 | 9473 | 9396 | 0.00 | 0.628 |
| 3 | 456,617,313 | 9471 | 9344 | 0.00 | 0.608 |
| 4 | 498,223,201,464 | 9513 | 9335 | 0.00 | 0.612 |
| 5 | 100,356,162,552,216 | 9562 | 9333 | 0.00 | 0.779 |

# Supplementary Table 4. Classification probabilities for most likely class membership by Latent Class

|  | **Most Likely Latent Class Membership** | | | |
| --- | --- | --- | --- | --- |
| **Latent Class** | **Stable Low**  **(n=498)** | **Stable High**  **(n=223)** | **Downwardly Mobile**  **(n =201)** | **Upwardly Mobile**  **(n =464)** |
| Stable Low | 0.862 | 0.001 | 0.053 | 0.083 |
| Stable High | 0.007 | 0.752 | 0.074 | 0.166 |
| Downwardly Mobile | 0.139 | 0.083 | 0.637 | 0.141 |
| Upwardly Mobile | 0.095 | 0.073 | 0.062 | 0.77 |

Probability of classification to ‘most likely class’ given membership in a ‘latent class’.

# Supplementary Table 5. Membership in latent classes of relative wealth mobility by relative wealth tertiles over the life course (n= 1387)

|  | **Pooled** | **Stable Low**  **(n=498)** | **Stable High**  **(n=223)** | **Downwardly Mobile**  **(n =201)** | **Upwardly Mobile**  **(n =464)** |
| --- | --- | --- | --- | --- | --- |
| 1967-75 Medium | 34.0% | 37.6% | 36.8% | 7.96% | 40.1% |
| High | 32.5% | 15.1% | 54.7% | 92.0% | 14.7% |
| 1987 Medium | 23.1% | 24.3% | 6.73% | 25.9% | 28.4% |
| High | 22.0% | 6.83% | 63.7% | 39.8% | 10.6% |
| *Missing* | 32.6% | 32.1% | 29.6% | 34.3% | 33.8% |
| 2002 Medium | 20.2% | 19.9% | 6.73% | 34.8% | 20.7% |
| High | 19.5% | 1.41% | 39.9% | 10.4% | 33.0% |
| *Missing* | 40.8% | 33.7% | 52.0% | 45.8% | 40.7% |
| 2015-18 Medium | 33.8% | 25.9% | 8.52% | 58.2% | 44.0% |
| High | 33.0% | 0.00% | 85.7% | 7.96% | 54.1% |

Categorical variables are displayed as percentage (%).

# Supplementary Table 6. Coefficients from regression with inverse probability weights^1^ for association of class membership with measures^2^ of health and human capital in middle adulthood (2015-18)

| ***Relative to Stable Low*** | **Height (cm)** | **Body mass index (kg/m^2^)** | **WHO SRQ-20** | **Ravens progressive matrices** |
| --- | --- | --- | --- | --- |
| Stable Low | Ref = 0.00 | Ref = 0.00 | Ref = 0.00 | Ref = 0.00 |
| Stable High | 3.13 (1.81, 4.45) | 1.39 (0.13, 2.65) | -0.67 (-1.51, 0.17) | 2.36 (1.33, 3.38) |
| Downwardly Mobile | 1.74 (0.7, 2.78) | 0.55 (-0.48, 1.57) | 0.43 (-0.31, 1.17) | 0.07 (-0.78, 0.92) |
| Upwardly Mobile | 1.62 (0.79, 2.44) | 1.32 (0.58, 2.06) | -0.87 (-1.37, -0.37) | 0.81 (0.2, 1.41) |
| ***Relative to Stable High*** |  |  |  |  |
| Stable Low | -3.13 (-4.45, -1.81) | -1.39 (-2.65, -0.13) | 0.67 (-0.17, 1.51) | -2.36 (-3.38, -1.33) |
| Stable High | Ref = 0.00 | Ref = 0.00 | Ref = 0.00 | Ref = 0.00 |
| Downwardly Mobile | -1.4 (-2.67, -0.12) | -0.84 (-1.98, 0.29) | 1.10 (0.30, 1.91) | -2.28 (-3.35, -1.21) |
| Upwardly Mobile | -1.51 (-2.6, -0.43) | -0.07 (-1.11, 0.97) | -0.2 (-0.89, 0.49) | -1.55 (-2.55, -0.55) |

Associations are adjusted for sex

1 Standardized IPW for being alive (maternal schooling, atole supplementation, exposure during first 1000 days, sex, birth year, wealth in 1967 or 1975) and reporting outcome (same as for being alive, additionally with wealth in 2015-18);

2 Height data available for 1,144 individuals; BMI data available for 1,138 individuals; SRQ-20 data available for 1,264 individuals; Ravens data available for 1,330 individuals.

# Supplementary Table 7. Coefficients from regression without auxiliary covariates in multiple imputation and with inverse probability weights for association of life course wealth gains with health outcomes in middle adulthood

|  | **Body mass index (kg/m^2^)** | **WHO SRQ-20** | **Ravens progressive matrices** |
| --- | --- | --- | --- |
| ***MI without Auxiliary covariates*^1^** |  |  |  |
| Wealth in 1967-75 | 0.04 (-0.24, 0.33) | -0.01 (-0.22, 0.19) | 0.09 (-0.16, 0.35) |
| Conditional Wealth 1987 | 0.11 (-0.11, 0.34) | 0.08 (-0.10, 0.25) | 0.36 (0.14, 0.58) |
| Conditional Wealth 2002 | 0.36 (0.04, 0.68) | -0.25 (-0.45, -0.05) | 0.35 (0.07, 0.64) |
| Conditional Wealth 2015-18 | 0.58 (0.25, 0.91) | -0.38 (-0.61, -0.16) | 0.49 (0.19, 0.78) |
| ***Inverse Probability Weights for reporting outcome^2^*** |  |  |  |
| Wealth in 1967-75 | 0.01 (-0.30, 0.32) | 0.01 (-0.20, 0.22) | -0.00 (-0.27, 0.27) |
| Conditional Wealth 1987 | 0.07 (-0.21, 0.35) | 0.08 (-0.11, 0.27) | 0.35 (0.13, 0.57) |
| Conditional Wealth 2002 | 0.27 (-0.11, 0.65) | -0.28 (-0.52, -0.05) | 0.35 (0.07, 0.62) |
| Conditional Wealth 2015-18 | 0.57 (0.24, 0.91) | -0.39 (-0.62, -0.15) | 0.50 (0.24, 0.76) |
| ***Inverse Probability Weights for being alive and reporting outcome^3^*** |  |  |  |
| Wealth in 1967-75 | 0.02 (-0.29, 0.33) | 0.02 (-0.19, 0.23) | 0.01 (-0.26, 0.28) |
| Conditional Wealth 1987 | 0.05 (-0.25, 0.34) | 0.05 (-0.15, 0.24) | 0.32 (0.09, 0.54) |
| Conditional Wealth 2002 | 0.31 (-0.06, 0.69) | -0.30 (-0.53, -0.06) | 0.32 (0.04, 0.61) |
| Conditional Wealth 2015-18 | 0.62 (0.28, 0.97) | -0.43 (-0.67, -0.19) | 0.47 (0.19, 0.74) |

Wealth is estimated from temporally harmonized index of assets and housing characteristics; all associations (change per 1 z-score in wealth) are adjusted for maternal schooling, assignment to atole supplementation group, exposure during first 1000 days, year of birth, sex, attained schooling and rural residence in adulthood.

1 Auxiliary covariates (atole supplementation, exposure during first 1000 days, sex, birth year, attained schooling and rural residence in adulthood) used only for maternal schooling;

2 Standardized IPW for for reporting outcome (maternal schooling, atole supplementation, sex, exposure during first 1000 days, birth year, attained schooling, rural residence in adulthood and wealth in 1967-75, 1987, 2002 and 2015-18)

3 Standardized IPW for being alive (maternal schooling, maternal age, atole supplementation, sex, exposure during first 1000 days, birth year, wealth in 1967-75) times IPW for reporting outcome

# Supplementary Table 8. Coefficients from zero-inflated poisson regression for association of wealth mobility with WHO SRQ-20

|  | **Risk Ratio (95% CI)** |
| --- | --- |
| ***Relative wealth mobility^1^*** |  |
| Stable Low | Ref = 1.00 |
| Stable High | 0.85 (0.72, 1.01) |
| Downwardly Mobile | 1.08 (0.95, 1.22) |
| Upwardly Mobile | 0.78 (0.70, 0.86) |
| ***Absolute wealth gains (z-scores)^2^*** |  |
| Wealth in 1967-75 | 1.01 (0.96, 1.06) |
| Conditional Wealth 1987 | 1.00 (0.96, 1.05) |
| Conditional Wealth 2002 | 0.94 (0.90, 0.98) |
| Conditional Wealth 2015-18 | 0.91 (0.87, 0.95) |

Zero-inflation was adjusted for sex.

^1^ Main terms were adjusted for maternal schooling, assignment to supplementation group, exposure during first 1000 days, year of birth, sex and attained schooling.

^2^Main terms model with absolute wealth associations (change per 1 z-score in wealth) were adjusted for maternal schooling, assignment to supplementation group, exposure during first 1000 days, year of birth, sex, attained schooling and rural residence in adulthood.

# Supplementary Table 9. Coefficients for association of absolute wealth gains with health in middle adulthood after stratifying by year at which early life wealth data was collected (n = 1386)

|  | **Body mass index (kg/m^2^)** | **WHO SRQ-20** | **Ravens progressive matrices** |
| --- | --- | --- | --- |
| ***Collected in 1967***  ***(n = 716)*** | **n = 594** | **n = 651** | **n = 682** |
| Wealth in 1967 | 0.34  (-0.14, 0.83) | 0.40  (0.05, 0.75) | 0.51  (0.09, 0.93) |
| Conditional Wealth 1987 | 0.04  (-0.29, 0.37) | -0.08  (-0.37, 0.21) | 0.13  (-0.18, 0.45) |
| Conditional Wealth 2002 | 0.56  (0.13, 0.99) | -0.31  (-0.60, -0.03) | 0.17  (-0.16, 0.50) |
| Conditional Wealth 2015-18 | 0.74  (0.29, 1.19) | -0.50  (-0.82, -0.19) | 0.34  (-0.03, 0.72) |
| ***Collected in 1975***  ***(n = 670)*** | **n = 544** | **n = 613** | **n = 648** |
| Wealth in 1975 | -0.11  (-0.48, 0.25) | -0.15  (-0.41, 0.10) | -0.04  (-0.38, 0.29) |
| Conditional Wealth 1987 | 0.14  (-0.23, 0.51) | 0.37  (0.13, 0.61) | 0.71  (0.40, 1.02) |
| Conditional Wealth 2002 | -0.00  (-0.49, 0.48) | -0.09  (-0.41, 0.24) | 0.61  (0.19, 1.04) |
| Conditional Wealth 2015-18 | 0.44  (-0.04, 0.92) | -0.28  (-0.60, 0.05) | 0.71  (0.28, 1.13) |

Wealth is estimated from temporally harmonized index of assets and housing characteristics; all associations (change per 1 z-score in wealth) are adjusted for maternal schooling, assignment to atole supplementation group, exposure during first 1000 days, year of birth, sex, attained schooling and rural residence. Coefficients are significantly different (p < 0.05) between two strata for Wealth in 1967 (SRQ-20, Ravens Progressive Matrices) and Conditional Wealth 1987 (SRQ-20, Ravens Progressive Matrices).
